# Mandated Substance Use Disorder Treatment in Qatar: An Innovative Model of Care

**DOI:** 10.1192/bjo.2025.10401

**Published:** 2025-06-20

**Authors:** Suhair Mohammed Yousuf, Nirvana Swamy Kudlur Chandrappa, Faycal Walid Ikhlef, Wesam S Smidi, Majid Ali Y. A. Al Abdulla

**Affiliations:** Mental Health Services - Hamad Medical Corporation, Doha, Qatar

## Abstract

**Aims:** Qatar has struggled with substance use disorders among its population. Qatar has maintained a relative political and social stability, which has informed a dramatic restructuring of its health and social care services with emphasis on being led by international best practice and primacy of patient rights. However, the rehabilitative model for substance use, which Qatar has placed emphasis on so far, has been based upon voluntary engagement of people who use substances. This has led to lack of provision of care to a significant proportion of patients with substance use disorders in addition to system-wide disagreements around models of care.

**Methods:** This study employed a retrospective patient record review of 163 patients admitted to the Umm Slal Treatment and Rehabilitation Center between January 2022 and October 2023. The data were systematically analysed to evaluate the effectiveness of the innovative Recovery Journey model.

**Results:** The majority of patients (61.3%) were aged 20–29, with 54% unemployed or students. Methamphetamine (77.3%) and cannabis (76.1%) were the most commonly used substances. Notable comorbidities included drug-induced psychosis (29.4%) and depression (19.5%). Most patients (90%) had previous treatment encounters. The Recovery Journey model, consisting of court-mandated detoxification and stabilization, residential rehabilitation, and community based continuing care, facilitated treatment completion for 91 out of 149 patients advancing from detoxification to rehabilitation. Challenges included managing complex co-occurring disorders and aligning multidisciplinary teamworking efforts.

**Conclusion:** The innovative Recovery Journey model at the Umm Slal Treatment and Rehabilitation Center demonstrated promising results in treating individuals with substance use disorders. While initial outcomes are encouraging, challenges related to stakeholder engagement, treatment adherence, and post-discharge care remain. This model emphasizes the importance of balancing directed care with patient autonomy and may serve as a framework for similar initiatives in the region. Further research into and adaptation of cultural contexts are essential for optimizing treatment outcomes.

